# Development of a highly sensitive method for detection of FLT3D835Y

**DOI:** 10.1186/s40364-020-00210-7

**Published:** 2020-08-12

**Authors:** Yao Guo, Honghua Sun, Dengyang Zhang, Yuming Zhao, Mingxia Shi, Ming Yang, Shu Xing, Xueqi Fu, Ting Bin, Bo Lu, Shunjie Wu, Xiaojun Xu, Xuesong Xu, Yun Chen, Zhizhuang Joe Zhao

**Affiliations:** 1 Edmond H. Fischer Translational Medical Research Laboratory, Scientific Research Center, The Seventh Affiliated Hospital, Sun Yat-sen University, Shenzhen, 518107 Guangdong China; 2 Department of Pathology, University of Oklahoma Health Sciences Center, 940 Stanton L. Young Blvd., BMSB 451, Oklahoma City, OK 73104 USA; 3 Clinical laboratory, The Seventh Affiliated Hospital of Sun Yat-sen University, Shenzhen, 518107 Guangdong China; 4 Department of Hematology, the First Affiliated Hospital of Kunming Medical University, Hematology Research Center of Yunnan Province, Kunming, 650032 China; 5 College of Life Sciences, Jilin University, Changchun, Jilin, 130012 China; 6 Department of Hematology, The Seventh Affiliated Hospital, Sun Yat-sen University, Shenzhen, China, Shenzhen, 518107 Guangdong China; 7 Clinical Laboratory of China-Japan Union Hospital, Jilin University, Changchun, Jilin, 130033 China

**Keywords:** Tyrosine kinase, Acute myeloid leukemia, FLT3-TKD, Detection of mutations

## Abstract

**Background:**

Acute myeloid leukemia (AML) is a malignant hematological neoplasm of myeloid progenitor cells. Mutations of FLT3 in its tyrosine kinase domain (FLT3-TKD) are found in ~ 8% of patients with AML, with D835Y as the most common substitution. This mutation activates survival signals that drives the disease and is resistant to the first generation FLT3 inhibitors. Development of a highly sensitive method to detect FLT3D835Y is important to direct therapeutic options, predict prognosis, and monitor minimal residual disease in patients with AML.

**Methods and results:**

In the present study, we developed a highly sensitive FLT3D835Y detection method by using the restriction fragment nested allele-specific PCR technique. The method consists of three steps: 1) initial amplification of DNA samples with PCR primers surrounding the FLT3D835Y mutation site, 2) digestion of the PCR products with restriction enzyme EcoRV that only cleaves the wild type allele, and 3) detection of FLT3D835Y by allele-specific PCR with nested primers. We were able to detect FLT3D835Y with a sensitivity of 0.001% by using purified plasmid DNAs and blood cell DNAs containing known proportions of FLT3D835Y. We analyzed blood cell DNA samples from 64 patients with AML and found six FLT3D835Y-positive cases, two of which could not be detected by conventional DNA sequencing methods. Importantly, the method was able to detect FLT3D835Y in a sample collected from a relapsed patient while the patient was in complete remission with negative MRD determined by flow cytometry. Therefore, our RFN-AS-PCR detected MRD after treatment that was missed by flow cytometry and Sanger DNA sequencing, by conventional methods.

**Conclusions:**

We have developed a simple and highly sensitive method that will allow for detection of FLT3D835Y at a very low level. This method may have major clinical implications for treatment of AML.

## Background

Acute myeloid leukemia (AML) is the most frequent form of leukemia in adults, representing approximately one third of all leukemia cases in the United States [1–3]. It is featured by the presence of myeloblasts in marrow and/or peripheral blood that impair normal hematopoiesis [4–6]. Although significant progress has been made in treatment of patients with a favorable prognosis [7–10], the overall survival of the disease remains poor [11].

To date, several molecular lesions have been identified in patients with AML [12–14]. Among them, FMS-like tyrosine kinase 3 (FLT3) is one of the most frequently mutated genes, found in around one-third of patients [9, 15, 16]. FLT3 is a receptor tyrosine kinase that has a crucial role in the development of hematopoietic progenitor cells. Gain-of-function mutations of FLT3 include internal tandem duplication (FLT3-ITD) and point mutations in the kinase domain (FLT3-TKD), each of which could lead to over-activated signals for proliferation and survival of leukemia cells [2, 17]. Studies have validated that both FLT3-ITD and FLT3D835Y are driver mutations in AML that cause myeloid neoplasms in murine models [18, 19], FLT3-ITD occurs in about 25% of AML patients and represents an unfavorable prognosis unless nucleophosmin 1 (NPM1) mutations are present [20, 21]. FLT3-TKD mainly includes the FLT3D835Y substitution and is found in ~ 7% of patients with AML [20, 21], FLT3 mutations could serve as important diagnostic and prognostic markers for AML. Elimination of cells carrying mutant FLT3 could be used to evaluate the clinical response and minimal residual disease (MRD) of patients during treatment. To date, several methods to detect FLT3-ITD have been developed [22], but convenient and sensitive methods to detect FLT3-TKD are still needed.

Current methods for FLT3-TKD genotyping include Sanger sequencing, restriction enzyme fragmentation, next generation sequencing, real-time polymerase chain reaction (real-time PCR), and denaturing high performance liquid chromatography [19, 23–26]. These methods have reported sensitivities ranging from 0.3 to 5%, and each has its own advantages and pitfalls. Some are not sensitive enough to detect MRD while others are sensitive but give nonspecific false positives. Also, some of these methods are labor-intensive and time-consuming, and they may require specialized or costly equipment and reagents [19, 25]. A more reliable and more sensitive method is still needed for the detection of mutant FLT3. In the present study, we developed a simple and highly sensitive method to detect FLT3D835Y based on the restriction fragment nested allele-specific PCR (RFN-AS-PCR) technique.

## Methods

### Sample collection and DNA extraction

Blood samples were collected from the clinical laboratory of the Seventh Affiliated Hospital of Sun Yat-sen University under an approved Institutional Review Board protocol. Genomic DNAs were purified by using the TaKaRa MiniBEST Universal Genomic DNA Extraction Kit Ver.5.0 (TaKaRa, Shiga, Japan). For each PCR reaction described below, around 1 μg total DNA was used.

### FLT3 and FLT3D835Y plasmid DNA standards

An 856-bp DNA fragment covering the D835 region of FLT3 was amplified from genomic DNA with primers F1_5 (5′- AGAAGCCGCACAAAGAAC) and F1_3 (5′ - CACCCAGCCAATTCACTC) and then cloned into the pGem-T vector (Promega, WI, USA). Plasmid DNAs were purified from *E. coli* cells by using the TaKaRa MiniBEST Plasmid Purification Kit Ver.4.0 (TaKaRa, Shiga, Japan). The plasmid containing FLT3D835Y was generated by PCR using Q5® Site-Directed Mutagenesis Kit (NEB, MA, USA) with primers 5′ - ATTGGCTCGAtATATCATGAGTG and 5′ – CCAAAGTCACATATCTTCAC. Purified FLT3 and FLT3D835Y plasmid DNAs were sequencing verified and mixed at different proportions and diluted to 20 μg/ml, and 1 μl of the DNA sample mixtures were used for PCR analysis described below. DNA concentrations were determined by measuring absorbance at 260 nm.

### Initial PCR, EcoRV restriction enzyme digestion, and nested AS-PCR

**Fig. 1:**
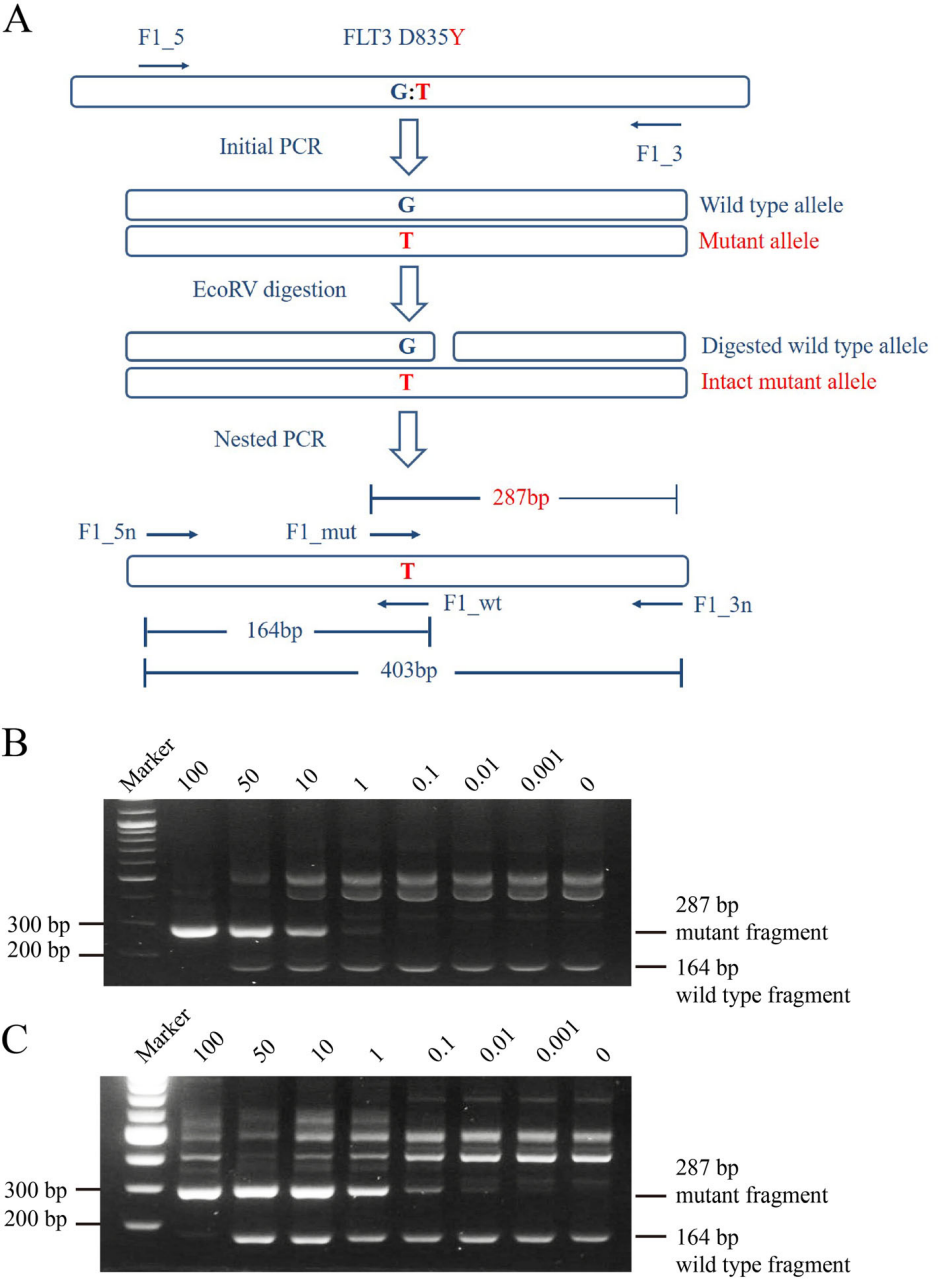
Development of RFN-AS-PCR for detection of FLT3D835Y. **a**. Schematic illustration of the RFN-AS-PCR method. **b** and **c** The sensitivities of nested AS-PCR and RFN-AS-PCR methods were determined by using purified plasmid DNAs. Mixtures of FLT3 plasmid DNAs containing the indicated percentages of the FLT3D835Y mutant were amplified with primers F1_5 and F1_3. The PCR products were left undigested (**b**) or digested with restriction enzyme EcoRV (**c**) and then subjected to nested AS-PCR analyses with a primer mixture containing F1_5n, F1_3n, F1_mut, and F1_wt. The final PCR products were analyzed on 3% agarose gel, and DNA bands were visualized by staining with SYBR green. The positions of wild type FLT3- and mutant FLT3D835Y-specfic products are indicated. The higher molecular weight bands shared by both wild type FLT3 and mutant FLT3D835Y are products of primer pairs F1_5n/F1_3n (403 bp), F1_5/F1_3, F1_5n/F1_3, and/or F1_5/F1_3n

The method of RFN-AS-PCR was described previously [27]. A schematic illustration is provided in Fig. 1a. In brief, isolated plasmid or blood cell DNAs were used as a template for initial PCR with primers F1_5 and F1_3. The PCR was run with Taq DNA polymerase for 35 cycles with each cycle consisting of 95 °C for 30 s, 60 °C for 30 s, and 72 °C for 1 min. The PCR products were then digested in a 10 μl reaction mixture containing 1 μl PCR products and 0.4 unit of EcoRV (NEB) at 37 °C for 1 h. The digested PCR products were further subjected to AS-PCR with nested primers F1_5n (5′ - GCACTCCAGGATAATACAC) and F1_3n (5′ - AGCCCAAGGACAGATGTGATG) and allele specific primers F1_mut (5′ - ATATGTGACTTTGGATTGGCTCTAT) and F1_wt (5′ - CATAGTTGGAATCACTCATGATCTC). The PCR was run for 35 cycles with each cycle consisting of 95 °C for 30 s, 55 °C for 30 s, and 72 °C for 30 s. The final PCR products were resolved on 3% agarose and visualized with SYBR green (Thermo Fisher Scientific, USA) staining. To ensure no cross-contamination occurred, control experiments with water replacing DNA samples were routinely performed and filter tips were used throughout. For sequencing verification, PCR products were gel-purified with TaKaRa MiniBEST Agarose Gel DNA Extraction Kit Ver.4.0 (TaKaRa, Shiga, Japan) and then analyzed by using an ABI3730 capillary sequencer (Genewiz, Suzhou, China). Each of the above experiments was repeated at least three times with consistent results.

### Detection of MRD with flow cytometry

Bone marrow samples from patient No.11 during the remission and relapse stages were analyzed by KingMed Diagnostics (Guangzhou, China).

## Results

### Development of restriction fragment nested allele-specific PCR (RFN-AS-PCR) for detection of FLT3D835Y

The FLT3D835Y mutation corresponds to c.2503G > T in DNA and disrupts an EcoRV restriction site (GATATC to TATATC). This provides a convenient way for detection of FLT3D835Y using the so-called restriction fragment length polymorphism technique that has a sensitivity of around 1%. On the other hand, allele-specific PCR is another commonly used method for detection of DNA point mutations with a sensitivity of around 1%. To enhance the sensitivity and specificity, we developed the method by combining restriction fragment length polymorphism with allele-specific PCR, and designated the method restriction fragment nested allele-specific PCR (RFN-AS-PCR). A schematic diagram of the procedure is illustrated in Fig. 1a. We first employed purified plasmid FLT3 and FLT3D835Y DNAs to determine the sensitivity of the method. The standards were created using a mixture of the two purified plasmid DNAs. Our data demonstrated that nested AS-PCR without EcoRV digestion had a sensitivity of 1% (Fig. 1b). With the EcoRV digestion step introduced, the RFN-AS-PCR technique increased the detection limit to 0.001% (Fig. 1c).

### Validation of the RFN-AS-PCR method using AML patient samples

**Fig. 2:**
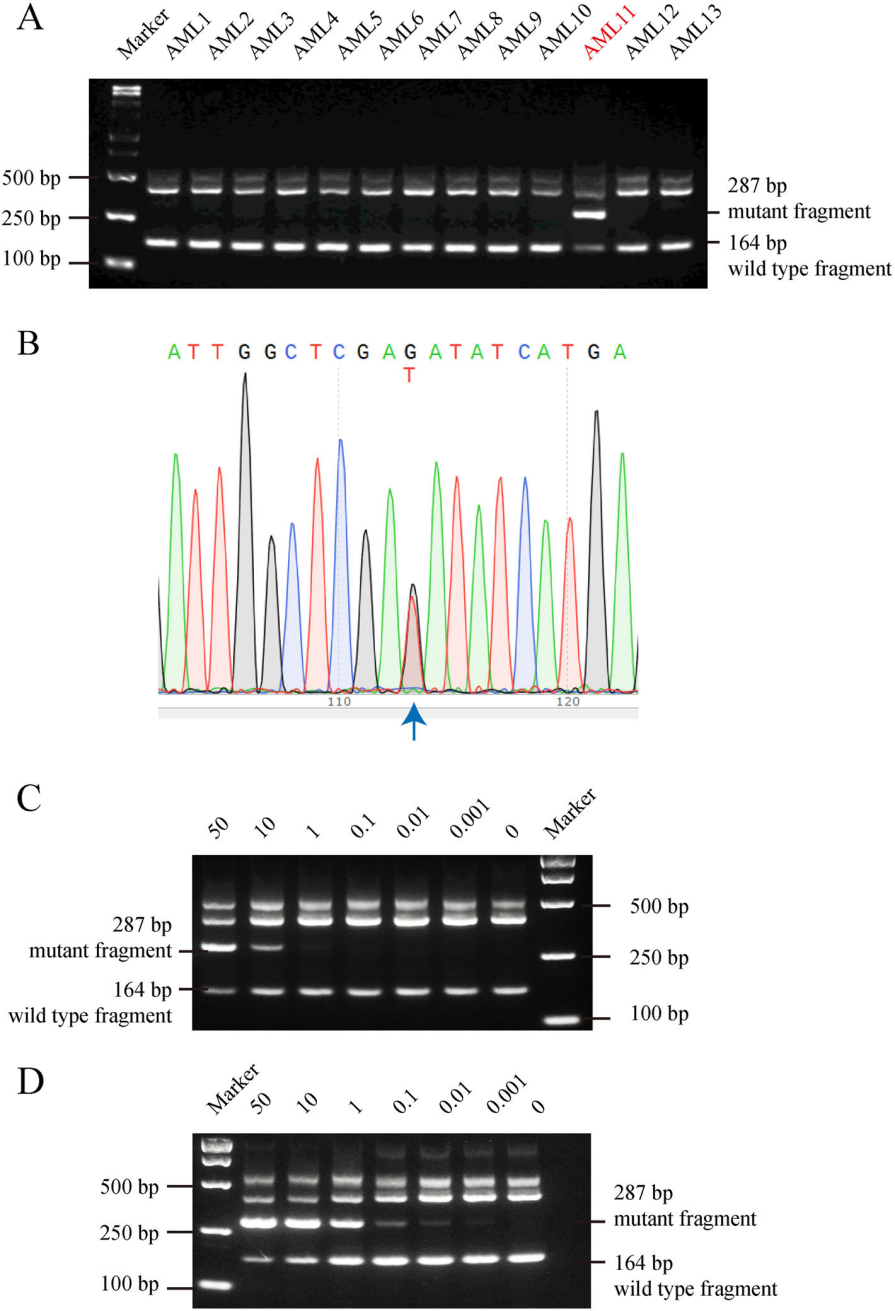
Validation of the RFN-AS-PCR method by using mixtures of DNA samples from FLT3D835Y mutant and wild type AML blood samples. **a** Screening of 13 AML blood samples by using RFN-AS-PCR identified AML11 as a FLT3D835Y-positive case. **b** Verification of FLT3D835Y positivity in AML11 by Sanger sequencing. **c** and **d** Blood cell DNAs from AML11 and a patient with wild type FLT3 were mixed in the indicated proportions. AS-PCR was performed without EcoRV digestion (panel **c**) or with EcoRV digestion (panel **d**). Note that the EcoRV digestion increased the detection sensitivity from 1 to 0.001%

We further validated the method by using a FLT3D835Y-positive AML patient blood DNA sample identified by our RFN-AS-PCR method (Fig. 2a). Sanger sequencing revealed that this particular sample (AML11) had a mutation rate of around 50% (Fig. 2b). We then mixed in various proportions of this FLT3D835Y-positive DNA sample and a FLT3-wild type blood DNA sample to create standards of genomic DNAs. A total of 1 μg of the DNA mixtures was used for the initial PCR, followed by nested AS-PCR with or without prior EcoRV digestion. Consistent with results obtained using standard plasmid DNA samples described above, nested AS-PCR alone was able to detect mixtures containing 1% of FLT3D835Y (Fig. 2c), while with the EcoRV digestion step, RFN-AS-PCR increased the detection sensitivity to 0.001% (Fig. 2d).

### Screening of AML samples for FLT3D835Y by RFN-AS-PCR

**Fig. 3:**
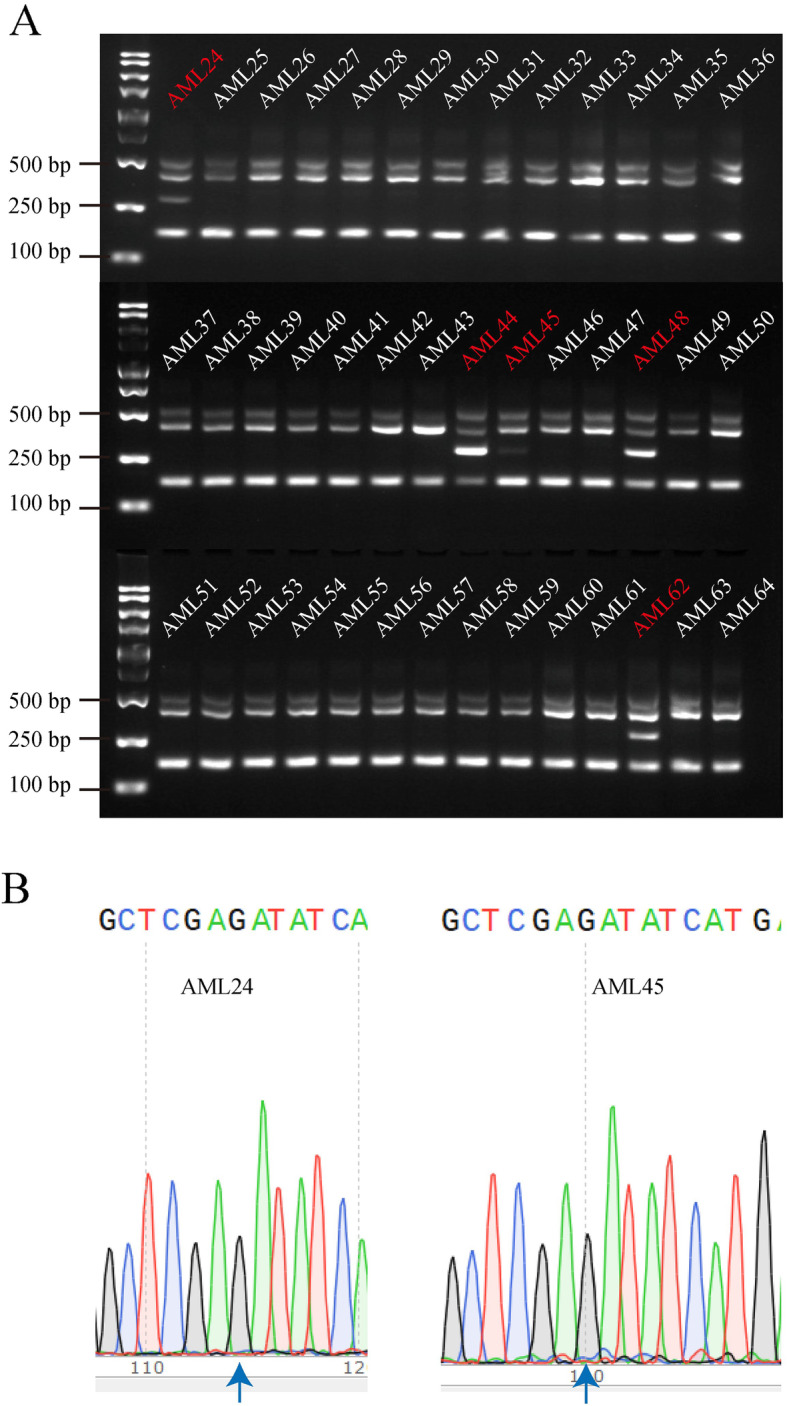
Screening of AML samples for identification of FLT3D835Y by using RFN-AS-PCR. **a** Detection of FLT3D835Y in 40 patients with AML by RFN-AS-PCR. **b** Failure of Sanger sequencing to detect FLT3D835Y in AML24 and AML45

**Fig. 4:**
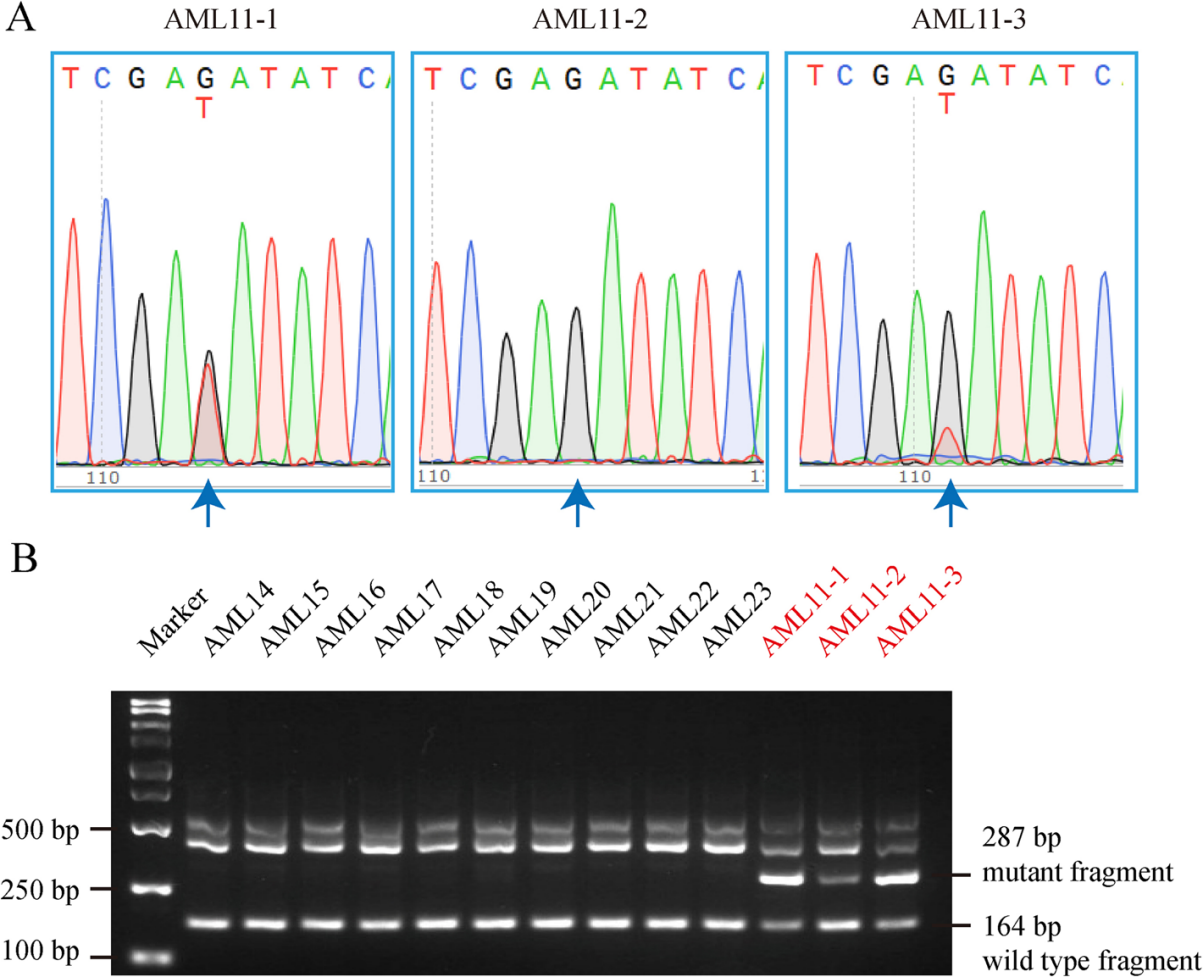
Detection of MRD by RFN-AS-PCR in an AML patient with FLT3D835Y. Blood DNA samples from an AML patient at initial diagnosis (AML11–1), in remission after treatment (AML11–2), and after relapse (AML11–3) were analyzed for FLT3D835Y using Sanger sequencing (**a**) and RFN-AS-PCR (**b**). Note that AML11–2 is FLT3D835Y-positive based on RFN-AS-PCR but negative according to Sanger sequencing. Ten additional FLT3D835Y-negative AML samples were analyzed in parallel

We then applied the RFN-AS-PCR method to screen AML patients for FLT3D835Y positivity. Out of 64 samples collected from a cohort of 64 AML patients (see Figs. 2a, 3a, and 4a), we found 6 FLT3D835Y-positive cases, corresponding to a 9.4% rate, which is higher than the reported rate of all FLT3-TKD mutations in patients with AML of 3.8 to 7.7% [28–30]. Figure 3a shows data from 36 of these samples. The relative low sample size may contribute to this high mutation rate. However, we found two of the positive samples had a low level of mutation that was not detectable with Sanger sequencing (Fig. 3b). This partly explains the high percentage of samples with the FLT3D835Y mutation rate observed in this study. It should be pointed out that this a low level of FLT3D835Y may not be the primary cause of the AML phenotypes for patients when the samples were collected. It probably co-exists with other mutations (e.g., FLT3-ITD) and may be responsible for relapse and progression of the disease to some degree. This potentially explains why some FLT3-ITD-positive patients treated with the first-generation FLT3 inhibitors often relapse with appearance of FLT3D535Y.

### Detection of MRD by RFN-AS-PCR in an FLT3D835Y-positive AML patient in complete remission

We further applied the RFN-AS-PCR method to analyze DNA samples collected from a 69-year-old AML patient before and after treatment. The patient was determined to have the FLT3D835Y mutation by Sanger DNA sequencing of blood DNA samples (Fig. 4). After treatment with decitabine plus homoharringtonine, cytarabine, and G-CSF, complete remission was achieved with negative MRD determined by flow cytometry analysis and DNA sequencing. Unfortunately, the disease relapsed 2 weeks later with 18.4% myeloblasts in bone marrow cells and positive Sanger DNA sequencing results. However, when blood DNA samples were analyzed by using the RFN-AS-PCR method, FLT3D835Y was found in all samples including the one at remission despite a relatively weak signal. Our RFN-AS-PCR detected MRD after treatment that was missed by flow cytometry and Sanger DNA sequencing. Therefore, the RFN-AS-PCR method should be a more reliable method to detect MRD for FLT3D835Y-positive AML patients.

## Discussion

In the present study, we developed a highly sensitive RFN-AS-PCR method to detect FLT3D835Y by combining restriction enzyme digestion and AS-PCR. The method is simple, quick, and inexpensive, and it does not require specialized equipment and reagents. Our data show that the method has a sensitivity of up to 0.001% and is able to detect FLT3D835Y in samples that are not detectable with other conventional methods. With the RFN-AS-PCR method, we found that 9.4% (6/64) of AML were FLT3D835Y-positive, which is higher than the reported 3.8 to 7.7% rate for all combined FLT3-TKD mutations in AML [28]. The data suggest that FLT3D835Y is more common than previously thought.

Both FLT3-TKD and FLT3-ITD are gain-of-function of mutations that cause constitutive activation of FLT3 signaling. However, their activation mechanisms differ and so do their responses to FLT3 inhibitors [17]. In fact, several FLT3 inhibitors including sorafenib and quizartinib potently inhibit FLT3-ITD but are not effective toward FLT3-TKD [31]. Consequently, FLT3-TKD-positive AML patients are resistant to treatment with sorafenib and quizartinib. Furthermore, many FLT3-ITD-positive AML patients relapse with the appearance of FLT3-TKD mutations after initial response to FLT3 inhibitor treatment. A plausible explanation for this is that FLT3-TKD mutations co-exist with FLT3-ITD at a very low level and become prevalent after FLT3-ITD-positive leukemic cells are killed. The two AML cases with very low level of FLT3D835Y identified in this study may belong to this scenario. Therefore, the presence of FLT3D835Y should be used to guide treatment plans for patients with AML.

Detection of MRD has major implications for recommending therapies and predicting outcomes in the treatment of AML [32]. Current methods for assessing MRD include multicolor flow-cytometry (MFC), real-time PCR, and next-generation sequencing (NGS) [33]. Although NGS is capable of detecting multiple molecular lesions with the sensitivity level of around 1%, it is considered labor-intensive with a longer turnaround time compared with other methods [34]. The detection of residual leukemic cells by MFC relies on the detection of antigens that are absent or rare in normal bone marrow cells. It is fast and cost-effective, but immunophenotypes of leukemic cells could change during the treatment, which potentially contributes to inaccuracy of the method [35]. Currently, the occurrence of relapse in AML patients with negative MRD ranges roughly between 20 and 25%, reflecting a major problem with the sensitivity and accuracy of currently available MRD detection techniques [33]. Our RFN-AS-PCR for FLT3D835Y has the sensitivity of 0.001% that by far outperforms the existing methods. In this regard, our study provides a powerful tool to detect FLT3D835Y to support diagnosis, therapeutics, and MRD-monitoring for patients with AML.

## Conclusions

We have developed a simple and highly sensitive method that will allow for detection of FLT3D835Y at a very low level. This method may have major clinical implications for diagnosis and treatment of AML.

## Data Availability

All data generated or analysed during this study are included in this published article and its supplementary information files.

## Abbreviations

AML: acute myeloid leukemia
FLT3: FMS-like tyrosine kinase 3
FLT3-ITD: FLT3 internal tandem duplication
FLT3-TKD: FLT3 tyrosine kinase domain mutations
NPM1: nucleophosmin 1
PCR: polymerase chain reaction
MRD: minimal residual disease
RFN-AS-PCR: restriction fragment nested allele-specific PCR
MFC: multicolor flow-cytometry
NGS: next-generation sequencing

## References

[1] Saygin C, Carraway HE (2017). Emerging therapies for acute myeloid leukemia. J Hematol Oncol.

[2] Chen Y, Pan Y, Guo Y, Zhao W, Ho WT, Wang J (2017). Tyrosine kinase inhibitors targeting FLT3 in the treatment of acute myeloid leukemia. Stem Cell Investig..

[3] Yang X, Wang J (2018). Precision therapy for acute myeloid leukemia. J Hematol Oncol.

[4] Ding Y, Gao H, Zhang Q (2017). The biomarkers of leukemia stem cells in acute myeloid leukemia. Stem Cell Investig..

[5] Kumar B, Chen CC (2018). Acute myeloid leukemia remodels endosteal vascular niche into a leukemic niche. Stem Cell Investig..

[6] Hanmantgad M, Nog R, Seiter K (2017). Acute myeloid leukemia and fatal Scedosporium prolificans sepsis after eculizumab treatment for paroxysmal nocturnal hemoglobinuria: a case report. Stem Cell Investig..

[7] Potluri S, Coleman D, Bonifer C (2018). Pharmacological inhibition of aberrant transcription factor complexes in inversion 16 acute myeloid leukemia. Stem Cell Investig.

[8] Ling Y, Xie Q, Zhang Z, Zhang H (2018). Protein kinase inhibitors for acute leukemia. Biomark Res.

[9] Wu M, Li C, Zhu X (2018). FLT3 inhibitors in acute myeloid leukemia. J Hematol Oncol.

[10] Lichtenegger FS, Krupka C, Haubner S, Kohnke T, Subklewe M (2017). Recent developments in immunotherapy of acute myeloid leukemia. J Hematol Oncol.

[11] Khwaja A, Bjorkholm M, Gale RE, Levine RL, Jordan CT, Ehninger G (2016). Acute myeloid leukaemia. Nat Rev Dis Primers.

[12] Hackl H, Astanina K, Wieser R (2017). Molecular and genetic alterations associated with therapy resistance and relapse of acute myeloid leukemia. J Hematol Oncol.

[13] Zhou J, Chng WJ (2017). Aberrant RNA splicing and mutations in spliceosome complex in acute myeloid leukemia. Stem Cell Investig..

[14] Gu R, Yang X, Wei H (2018). Molecular landscape and targeted therapy of acute myeloid leukemia. Biomark Res..

[15] Perl AE (2019). Availability of FLT3 inhibitors: how do we use them?. Blood..

[16] Wang ES (2019). Incorporating FLT3 inhibitors in the frontline treatment of FLT3 mutant acute myeloid leukemia. Best Pract Res Clin Haematol.

[17] Chen Y, Guo Y, Zhao W, Tina Ho WT, Fu X, Zhao ZJ (2016). Identification of an orally available compound with potent and broad FLT3 inhibition activity. Oncogene..

[18] Bailey E, Li L, Duffield AS, Ma HS, Huso DL, Small D (2013). FLT3/D835Y mutation knock-in mice display less aggressive disease compared with FLT3/internal tandem duplication (ITD) mice. Proc Natl Acad Sci U S A.

[19] Smith CC, Wang Q, Chin CS, Salerno S, Damon LE, Levis MJ (2012). Validation of ITD mutations in FLT3 as a therapeutic target in human acute myeloid leukaemia. Nature..

[20] Perry M, Bertoli S, Rocher C, Hayette S, Ducastelle S, Barraco F (2018). FLT3-TKD mutations associated with NPM1 mutations define a favorable-risk Group in Patients with Acute Myeloid Leukemia. Clin Lymphoma Myeloma Leuk.

[21] Boddu P, Kantarjian H, Borthakur G, Kadia T, Daver N, Pierce S (2017). Co-occurrence of FLT3-TKD and NPM1 mutations defines a highly favorable prognostic AML group. Blood Adv.

[22] Mezei ZA, Tornai D, Foldesi R, Madar L, Sumegi A, Papp M (2019). A DNA pool of FLT3-ITD positive DNA samples can be used efficiently for analytical evaluation of NGS-based FLT3-ITD quantitation - testing several different ITD sequences and rates, simultaneously. J Biotechnol.

[23] Banko P, Lee SY, Nagygyorgy V, Zrinyi M, Chae CH, Cho DH (2019). Technologies for circulating tumor cell separation from whole blood. J Hematol Oncol.

[24] Trinh PL, Pham Y (2018). Establishment of a multiplex PCR-based procedure for detection of Most common mutations in NPM1, FLT3 in acute myeloid leukemia patients. Ann Clin Lab Sci.

[25] Ali A, Gale RE, Shakoori AR (2017). Detection of FLT3/TKD and IDH1 mutations in Pakistani acute myeloid leukemia patients by denaturing HPLC. J Cell Biochem.

[26] Wang LH, Wang M, Zhou CL, Chen S, Zhang XW, Xing HY (2005). Detection of point mutation at second tyrosine kinase domain of FLT3 gene in acute myeloid leukemia. Zhonghua Xue Ye Xue Za Zhi.

[27] Zhao AH, Gao R, Zhao ZJ (2011). Development of a highly sensitive method for detection of JAK2V617F. J Hematol Oncol.

[28] Thiede C, Steudel C, Mohr B, Schaich M, Schakel U, Platzbecker U (2002). Analysis of FLT3-activating mutations in 979 patients with acute myelogenous leukemia: association with FAB subtypes and identification of subgroups with poor prognosis. Blood..

[29] Li L, Lyu XD, Mi RH, Ding J, Chen L, Wang Q (2013). Detection of NPM1, FLT3 and C-KIT mutations in acute myeloid leukemia and their prognostic analysis. Zhongguo Shi Yan Xue Ye Xue Za Zhi.

[30] Liu Y, Ke XY, Wang J, Wang JJ, Jing HM, Dong F (2018). Clinical characteristics and prognosis of acute myeloid leukemia with FLT3-ITD mutation. Zhongguo Shi Yan Xue Ye Xue Za Zhi..

[31] Daver N, Schlenk RF, Russell NH, Levis MJ (2019). Targeting FLT3 mutations in AML: review of current knowledge and evidence. Leukemia..

[32] Medeiros BC, Chan SM, Daver NG, Jonas BA, Pollyea DA (2019). Optimizing survival outcomes with post-remission therapy in acute myeloid leukemia. Am J Hematol.

[33] Voso MT, Ottone T, Lavorgna S, Venditti A, Maurillo L, Lo-Coco F (2019). MRD in AML: the role of new techniques. Front Oncol.

[34] Kohlmann A, Grossmann V, Nadarajah N, Haferlach T (2013). Next-generation sequencing - feasibility and practicality in haematology. Br J Haematol.

[35] Buccisano F, Maurillo L, Del Principe MI, Di Veroli A, De Bellis E, Biagi A (2018). Minimal residual disease as a biomarker for outcome prediction and therapy optimization in acute myeloid leukemia. Expert Rev Hematol.

